# Rho-Kinase II Inhibitory Potential of* Eurycoma longifolia* New Isolate for the Management of Erectile Dysfunction

**DOI:** 10.1155/2019/4341592

**Published:** 2019-05-15

**Authors:** Shahira M. Ezzat, Mona M. Okba, Marwa I. Ezzat, Nora M. Aborehab, Shanaz O. Mohamed

**Affiliations:** ^1^Pharmacognosy Department, Faculty of Pharmacy, Cairo University, Kasr El-Ainy Street, Cairo 11562, Egypt; ^2^Pharmacognosy Department, Faculty of Pharmacy, October University for Modern Sciences and Arts (MSA), 6^th^ October 12566, Egypt; ^3^Biochemistry Department, Faculty of Pharmacy, October University for Modern Sciences and Arts (MSA), 6th October 12566, Egypt; ^4^School of Pharmaceutical Sciences, Universiti Sains Malaysia, Malaysia

## Abstract

*Background. Eurycoma longifolia *Jack (Fam.: Simaroubaceae), known as Tongkat Ali (TA), has been known as a symbol of virility and sexual power. The aim of the study was to screen* E. longifolia* aqueous extract (AE) and isolates for ROCK-II inhibition.* Results.* The AE (1-10 *μ*g/ml) showed a significant inhibition for ROCK-II activity (62.8-81%) at* P* < 0.001 with an IC_50_ (651.1 ± 32.9 ng/ml) compared to Y-27632 ([(+)-(*R*)-*trans*-4-(1-aminoethyl)-*N*-(4-pyridyl)cyclohexanecarboxamide dihydrochloride]) (68.15-89.9 %) at same concentrations with an IC_50_ (192 ± 8.37 ng/ml). Chromatographic purification of the aqueous extract (AE) allowed the isolation of eight compounds; stigmasterol** T1**,* trans-*coniferyl aldehyde** T2,** scopoletin** T3**, eurycomalactone** T4**, 6*α*- hydroxyeurycomalactone** T5**, eurycomanone** T6**, eurycomanol** T7**, and eurycomanol-2-*O*-*β*-D-glucopyranoside** T8**. This is the first report for the isolation of** T1** and** T3** from* E. longifolia* and for the isolation of** T2** from genus* Eurycoma*. The isolates (at 10 *μ*g/ml) exhibited maximum inhibition % of ROCK-II 82.1 ± 0.63 (T2), 78.3 ± 0.38 (T6), 77.1 ± 0.11 (T3), 76.2 ± 3.53 (T4), 74.5 ± 1.27 (T5), 74.1 ± 2.97 (T7), 71.4 ± 2.54 (T8), and 60.3 ± 0.14 (T1), where the newly isolated compound* trans-*coniferyl aldehyde** T2 **showed the highest inhibitory activity among the tested isolated compounds and even higher than the total extract AE. The standard Y-27632 (10 *μ*g/ml) showed 89.9 ± 0.42 % inhibition for ROCK-II activity when compared to control at* P *< 0.0001.* Conclusion.* The traditional use of* E. longifolia* as aphrodisiac and for male sexual disorders might be in part due to the ROCK-II inhibitory potential.

## 1. Introduction

Libido refers to a fluctuating state of sexual desire [1]. The 21st century has seen the evolution of a lot of firms and clinics that claim to treat reduced libido in males [2]. Studies have reported a prevalence of the Hypoactive Sexual Desire Disorder (HSDD) in men between 1 and 20% [3]. It is estimated that 30-40% of people around the world experience lack of sexual interest for at least several months in any given year [2]. Nowadays, sexual desire is controlled by some external factors including psychiatric disorders as depression, some types of medications including antidepressants, some diseases as diabetes and hypothyroidism, social and interpersonal problems, and other conditions causing inhibited or decreased dopamine release, leading to sexual dysfunction, general lack of sexual desire, and decreased libido [1]. Alteration in libido also may be due to some biochemical messengers, such as levels of serum steroid hormone (mainly testosterone), feedback after sexual stimulation, and disturbances in the brain neurotransmitters [4]. Till this moment, the only available medicines indicated to increase male libido are some herbal drugs and hormonal therapy in cases of testosterone deficiency [5].

*E. longifolia* (Tongkat Ali, Genus: Eurycoma; Family: Simaroubaceae) is one of the most well-known tropical plants, indigenous to Southeast Asian countries like Vietnam, Malaysia, and Indonesia. It is known as ‘Tongkat Ali' where in Malaysia ‘Ali' refers to “walking stick” because this plant roots are twisted and long. The plant (particularly roots) has been traditionally used for reducing fever and fatigue and for its unique antimalarial, antipyretic, antiulcer, and its aphrodisiac properties. Body builders have been recently focusing on regular intake of its root extracts to improve muscular mass and strength [6–8].

A large number of phytochemicals have been detected and identified from E. longifolia roots including eurycomanone, eurycomaoside, eurycolactone, eurycomalactone, canthin-6-one alkaloids, quassinoid diterpenoids, *β*-carboline alkaloids, tirucallane-type triterpenes, biphenylneolignans, laurycolactone, and squalene derivatives [9, 10].* E. longifolia* has gained wide appreciation for its uniqueness in enhancing sexual power which was supported by some literature in experimental animals [11–15]. It has been utilized by Malaysian men for hundreds of years to enhance the quality and performance of sexual exercise [6, 7].

Around the world, there has been a gigantic increment in the utilization of this plant. There are about two hundred Tongkat Ali products, mostly focusing on the sexual enhancing properties. It is available either as crude root powder, in capsules blended with different aphrodisiac drugs, as an added substance blended with ginseng or coffee, or in other healthcare products as a substitute for ginseng [8].

Corpus cavernosum smooth muscle (CCSM) and penile arteries relaxation results in blood trapping in the penis leading to raised intracavernous pressure (ICP) which plays a pivotal role as penile erection [16].

RhoA and ROCK are found in different tissues in the body and responsible for regulating many functions. In spite of their presence in the neural and endothelial tissues of the human corpora, but their prominent effects are obvious in penile erection through modulation of cavernous sinusoidal and arteriolar smooth-muscle cells contractile state [17].

Although Tongkat Ali traditional use as an aphrodisiac herb is well-recognized, there is no sufficient information on the possible underlying mechanisms. Therefore, this study was designed to evaluate* E. longifolia *AE and isolated biophytochemicals potential in management of erectile dysfunction (ED).

## 2. Materials and Methods

### 2.1. Plant Material

The roots of* Eurycoma longifolia *Jack were obtained from HCA products Sdn Bhd. Spring 2015. The plant was kindly identified in the Forest Research Institute, Malaysia. A voucher specimen (5-09-2015) was kept in the herbarium of Pharmacognosy Department, Faculty of Pharmacy, Cairo University, Cairo, Egypt.

### 2.2. Preparation of the Aqueous Extract (AE)

The collected roots were washed with running water and then dried on an open surface and dried by exposure to sunlight for 1 or 2 days to ensure freedom of humidity. The dried roots were then chipped to 5 mm particles. The dried chipped roots (40 kg) were boiled with 200 liters of RO water (water purified with reverse osmosis) for 3 hours; the extract was concentrated in a rotary evaporator for 3 hours at 60°C to 20 liters. The extract was then dried in a spray dryer by heating for 6h and 30 min at a temperature of 120°C and yielded 1.6 kg powdered extract where the extract yield is 4%.

### 2.3. Rock-II Inhibition Assay

The assay was done as mentioned in ADP-Glo™ Kinase Assay (SER-THR KINASE SERIES: ROCK2 Kinase assay) (Promega, USA) and Y-27632 [(+)-(*R*)-*trans*-4-(1-aminoethyl)-*N*-(4-pyridyl)cyclohexanecarboxamide dihydrochloride] was used as standard drug; luminescence was recorded using Topotecan, USA, Spark 10 M, multimode microplate reader. A vehicle control for 5% DMSO was used in the assay to check the interference. Standard curve for ROCK-II enzyme was done (Figure 2). Serial dilution and IC_50_ of the AE was performed in triplicate.

### 2.4. Fractionation of the AE and Isolation of Its Major Phytochemicals

#### 2.4.1. General

Silica gel 60 (70 - 230 mesh ASTM; Fluka, Steinheim, Germany), Diaion HP-20 AG, Sephadex LH-20 (Pharmacia Fine Chemicals AB, Uppsala, Sweden), and reversed phase silica gel (RP-18) (70-230 mesh) for column chromatography (75-150 *μ*m, Mitsubishi Chemical Industries Co. Ltd). Thin-layer chromatography (TLC) (silica gel GF_254_ precoated plates- Fluka) was done using this solvent systems: S_a_:* n*-Hexane: ethyl acetate (7:3 v/v); S_b_; ethyl acetate-methanol-water-formic acid (10:1.6:1.2:1 v/v). Chromatograms detections were performed under UV light (at 254 and 366 nm) and sprayed by* p*-anisaldehyde sulphuric acid spray reagent. Bruker NMR was used for ^13^C-NMR (125 MHz) and ^1^H-NMR (400 MHz). The NMR spectra were observed in DMSO and CD_3_OD. Chemical shifts are given in* δ *(ppm) relative to internal standard TMS.

#### 2.4.2. Isolation of the Major Phytochemicals

For isolation of the major compounds, 500 grams of AE were suspended in 800 ml distilled water then defatted with methylene chloride (300 mLx 3). The organic and aqueous layers were separated. The organic layer was evaporated using rotary evaporator under reduced pressure at 40°C to yield 8 gm of methylene chloride residue (MeCl). The aqueous layer was kept for further fractionation.

MeCl (8 g) was fractionated over a silica gel column (80 g). Gradient elution was done using* n*-hexane-methylene chloride then methylene chloride-methanol mixtures. The polarity was increased by 10 % incriminations of methylene chloride in* n*-hexane every 50 ml till 100% methylene chloride then further 1% incriminations of methanol in methylene chloride till 7% methanol. Fractions (20 ml) were collected to obtain 60 fractions which were then monitored by TLC using solvent system (S_1_). Subfraction (60% methylene chloride in* n*-hexane) was washed with methanol to yield pure compound** T1** (white crystals, 25 mg). Subfraction (80% methylene chloride in* n*-hexane) was chromatographed over a silica gel column. The elution carried out using* n*-hexane-ethyl acetate (85:15 v/v). Similar fractions were pooled together to yield compound** T2** (white crystals, 20 mg). Fraction (1% methanol in methylene chloride) was chromatographed over a sephadex LH20 using methanol-water (7:3 v/v) as eluent to yield one compound** T3 **(yellowish white crystals, 34 mg). Fraction (6% methanol in methylene chloride) was chromatographed over a sephadex LH20 using* n*-butanol-isopropanol-water (4:1:5 v/v) as eluent to yield a fraction containing two major spots with minor impurities. This fraction was further purified by rechromatography over silica gel column. It was gradient eluted using* n*-hexane-ethyl acetate (10-30%) mixtures to yield two pure compounds** T4** (white crystals, 68 mg) and** T5 **(white crystals, 52 mg).

The defatted aqueous solution was chromatographed on diaion HP-20 AG (500 g) column. Elution was carried out with water, followed by methanol-water (50%), methanol-water (75%), and methanol (100%) to give four fractions (D1-D4), respectively. The solvent in each case was evaporated using rotary evaporator to yield solid residues weighing 154, 35, and 10 g, respectively. Methanol-water (50%) (D2) fraction (35g) was further fractionated over a silica gel (100 g) column where elution was carried out by* n*-hexane: ethyl acetate. Gradient elution was carried out by* n*-hexane-ethyl acetate and ethyl acetate-methanol-water mixtures. The polarity was increased by 10 % incriminations of ethyl acetate every 100 ml till 100% ethyl acetate then further incrimination of methanol (till 1.6%) and water (till 1.3%). Fractions (20 ml, each) were combined to give 60 fractions which were monitored by TLC using solvent systems (Sb). Subfraction (80% ethyl acetate in* n*-hexane) was fractionated over a silica (RP) column. The elution carried out using water-methanol as eluent. The fractions eluted with 10% and 20% methanol give compounds** T6** (white powder, 83 mg) and** T7 **(white powder, 90 mg), respectively. Subfraction (0.9% methanol, 0.3% water in ethyl acetate) was chromatographed over a sephadex column eluted with 50% methanol then silica gel column eluted with ethyl acetate-methanol (9:1 v/v) to give compound** T8 **(white crystals, 80 mg).

### 2.5. Rock-II Inhibition Assay

The assay was repeated as mentioned in Section 2.3 on the AE fractions and the isolates T1-T8.

The assay performance measure was used to validate the screening assay quality through calculation of Z-factor according to methodology of Zhang et al., 1999 [18].

### 2.6. Statistical Analysis

Enzyme inhibition by tested samples is expressed as mean ± SD and analyzed using Prism program version 6 (GraphPad Software, Inc., San Diego CA); comparisons among tested samples were carried out using one-way analysis of variance (ANOVA) followed by Bonferroni's multiple comparisons test.* P ‹* 0.05 was considered significant.

## 3. Results

### 3.1. Evaluation of AE Rock-II Inhibition Potential

Concentrations at (1-10 *μ*g/ml) of the AE and Y-27632 as a standard showed a significant inhibition for ROCK-II activity (62.8-81%). The inhibition of ROCK-II activity at* P* < 0.001 was recorded in Table 1. IC_50_ in ROCK-II inhibition assay of AE (651.1 ± 32.9 ng/ml) and Y-27632 were recorded in Table 2.

### 3.2. Fractionation of AE and Isolation of the Major Phytochemicals

**Figure 1:**
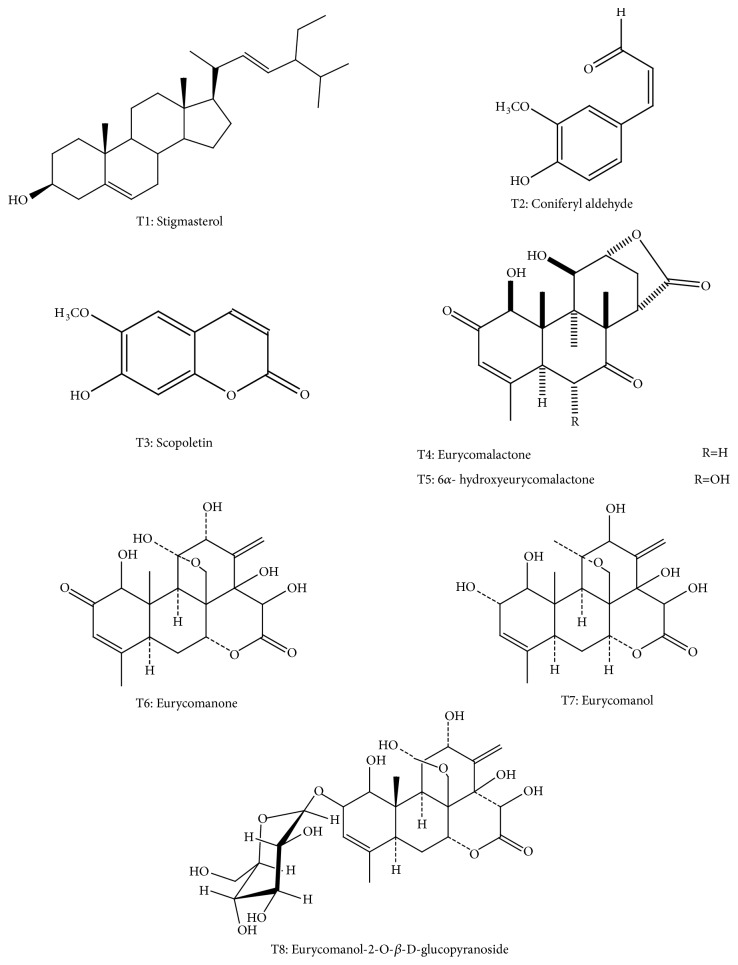
Structure of the isolated compounds (T1-8).

**Figure 2:**
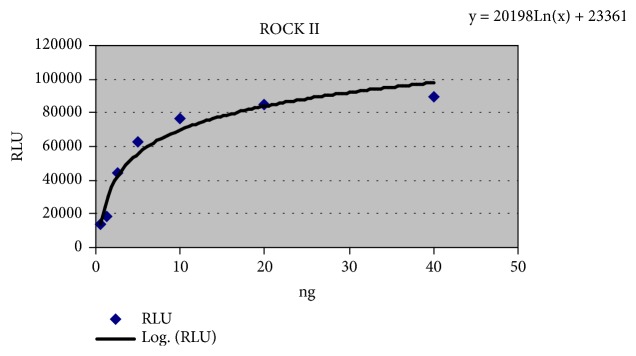
*ROCK-II enzyme standard curve.* x-axis represents the concentration from (40 ng/ml, 20 ng/ml, 10 ng/ml, 5 ng/ml, 2.5 ng/ml, 1.25 ng/ml and 0.625 ng/ml) and Y-axis represents ΔRLU.

Chromatographic fractionation of* E. longifolia* roots AE allowed the isolation of one sterol: stigmasterol,** T1**; a phenolic compound:* trans*-coniferyl aldehyde** T2**; one coumarin: scopoletin** T3**; and 5 known quassinoids namely eurycomalactone** T4**, 6*α*- hydroxyeurycomalactone** T5**, eurycomanone** T6**, eurycomanol** T7,** and eurycomanol-2-*O*-*β*-D-glycopyranoside** T8**. The isolated compounds were identified via their co-TLC comparison to authentic reference samples, physicochemical characters, and spectroscopic analysis and through comparing their 1D and 2D NMR data with the previously published data. ^1^H NMR and ^13^CNMR data of the isolated phytochemicals are presented in Tables S1 and S2 in the supplementary file. The structures of the isolated phytochemicals are shown in Figure 1.

### 3.3. Evaluation of Rock-II Inhibition Potential of AE Fractions and Isolates

All tested samples and Y-27632 standard at concentration range (0.01-10 *μ*g/ml) showed a significant inhibition for ROCK-II activity.

At dose 10 *μ*g/ml, MeCl, D1, D2, D3, D4 showed a maximum inhibition % of (86.3 ± 0.71), (90.1 ± 0.84), (86.1 ± 0.42), (90.25 ± 0.07), (87.05 ± 0.21), respectively.

The isolates (at 10 *μ*g/ml) exhibited maximum inhibition % of 82.1 ± 0.63 (T2), 78.3 ± 0.38 (T6), 77.1 ± 0.11 (T3), 76.2 ± 3.53 (T4), 74.5 ± 1.27 (T5), 74.1 ± 2.97 (T7), 71.4 ± 2.54 (T8), and 60.3 ± 0.14 (T1). The standard Y-27632 (10 *μ*g/ml) showed (89.9 ± 0.42) inhibition % for ROCK-II activity when compared to vehicle control at* P* < 0.0001. Nonsignificant difference was found between MeCl, D1, D2, D3, D4 at concentration 10 *μ*g/ml compared to Y-27632 at the same concentration against the inhibition of ROCK-II activity at* P* < 0.001 as presented in Table 1.

IC_50_ of ROCK-II inhibition assay of all tested AE fractions, isolates, and Y-27632 were recorded in Table 2.

Nonsignificant difference was found between MeCl, D1, D2, D3 and D4 with IC50 (162.8 ± 3.35, 105 ± 3.56, 153 ± 14.1, 91.1 ± 6.63, and 189.3 ± 21.9, respectively) compared to Y-27632 IC_50_ (192 ± 8.37); these fractions showed a prominent effect as the same effect as Y-27632 in ROCK-II inhibition.

The assay performance measure was evaluated by calculation of Z-factor which was equal to 0.802 which indicated that it is an excellent assay [18].

## 4. Discussion

*E. longifolia* roots AE has gained wide recognition for enhancing the virility and sexual prowess. It has been utilized by Malaysian men for hundreds of years to enhance the quality and performance of sexual exercises [6, 7]. Although traditional use of* E. longifolia* as an aphrodisiac herb is well-recognized, there is a paucity of information on the possible underlying mechanisms. Therefore, the present study aimed at substantiating the aphrodisiac activity of* E. longifolia.*

ROCK-II inhibition assay was performed using ADP-Glo™ Kinase Assay and Y-27632 was used as standard; this method was preferred more than ELISA technique due to the absence of several washing steps and false results that may happen due to the interference with horseradish peroxidase as the extracts have ant-oxidant activity.

Smooth-muscle contraction is regulated by the cytosolic Ca^2+^ concentration and by the calcium sensitivity of myofilaments. The major mechanism of Ca^2+^ sensitization of smooth-muscle contraction is achieved by the inhibition of the myosin light chain phosphatase (MLCP) that dephosphorylates the Myosin light chain in smooth muscle through RhoA/Rho-kinase pathway. The active, GTP bound form of the small GTPase RhoA activates a serine/threonine kinase, Rho-kinase (ROCK-II), which phosphorylates the regulatory subunit of MLCP and inhibits phosphatase activity leading to contraction of smooth muscle through Ca^2+^ sensitivity. MLCP converts the active phosphorylated myosin light chain (MLC) to inactive one so relaxation of the muscle occurs [19].

AE purification led to the isolation of eight compounds. Compound** T2** was isolated as needle crystals. Its ^1^HNMR spectrum showed three aromatic protons arranged in ABX system which was characterized by three doublets at *δ*_H_ 6.99 (1H, d,* J*=1.76 Hz), 6.88 (1H,d,* J*=8.16 Hz), and 7.04 (1H, dd,* J*=1.8,8.16 Hz) assigned to H-2, H-5, and H-6. In addition two* trans*-olefinic protons appeared at *δ*_H_ 7.31 (1H, d,* J*=15.8 Hz, H-7) and 6.49 (1H, dd,* J*=7.70,15.8 Hz, H-8) and an aldehydic group which appeared as a doublet at *δ*_H_ 9.56 (1H, d,* J*=7.70 Hz, H-9) and finally a methoxy group at *δ*_H_ 3.82 as a singlet. The coupling constants* J*_*7,8*_ and* J*_*8,9*_ indicated that Δ^7,8^ is trans and that CHO is linked to H-8; this was confirmed from HMBC correlations between 7.31 (1H, d,* J*=15.8 Hz, H-7) and C-8 at *δ*_C_ 126.4 and CHO at *δ*_C_ 193.6 and also the correlations of *δ*_H_ 9.56 (1H, d,* J*=7.70 Hz, H-9) with C-7 at *δ*_C_ 153.1 and C-8 at *δ*_C_ 126.4. The position of OCH_3_ at C-3 was deduced from long-range coupling between *δ*_H_ 3.82 and C-3 at *δ*_C_ 146.9. The assignments of carbons were deduced from ^1^H-^13^C correlations in HSQC. This compound was identified as* trans*-coniferyl aldehyde [20], which is isolated here for the first time from genus* Eurycoma*.

Compounds T1, T3-T8 spectral data were in agreement with the reported data of stigmasterol [21], scopoletin [22], eurycomalactone, 6*α*- hydroxyeurycomalactone [23], eurycomanone [23], eurycomanol [23], and eurycomanol-2-*O*-*β*-D-glycopyranoside [24]. This is the first report for the isolation of T1 and T3 from E. longifolia and for the isolation of T2 from genus* Eurycoma*.

Among the different doses used for the AE, MeCl, fractions and isolates, all of them exhibited more than 50% of ROCK-II inhibition at higher dose which indicate the use of this potent herbal drug in the management of erectile dysfunction. It is worth noting that maximum inhibition of ROCK-II was recorded for trans-coniferyl aldehyde (T2) 82.1% which is isolated from* Eurycoma* for the first time. Previous studies reported the potential antimutagenic, antioxidant [25], and anti-inflammatory properties of coniferyl aldehyde [26], but its effect on erectile dysfunction was not studied before.

Although the ROCK-II inhibitory potential of* E. longifolia *crude extract was studied once before [16], this is the first report to evaluate the inhibition activity of* E. longifolia *isolates (T1-T8) on ROCK-II that manage erectile dysfunction.

In a recent review about* E. longifolia* chemistry and evidence-based pharmacology, many* E. longifolia* isolated compounds pharmacological activities were reported [6]. All* E. longifolia* previously isolated compounds activities on ROCK-II that manage erectile dysfunction were not reported. Compounds isolated in our study exhibited other activities rather than improvement of sexual behavior; eurycomalactone and eurycomanol-2-*O*-*β*-D-glycopyranoside antimalarial activity [27], 6*α*- hydroxyeurycomalactone cytotoxic activity [28], and eurycomanol are the regulators of signaling pathways involved in proliferation, cell death, and inflammation [29], except for eurycomanone which was reported to improve sexual behavior by other mechanisms more than affecting erectile dysfunction.

Beside the herein reported potent effect in managing the erectile dysfunction, the positive effect of* E. longifolia* in the improvement of sexual behavior may be attributed to its active constituents such as quassinoids and in particular the major one, eurycomanone, which was isolated and identified in the present work. Eurycomanone was reported to induce testosterone production [6] and was also reported to enhance testosterone steroidogenesis at the Leydig cells through its inhibitory effect on the final step of transformation of testosterone to estrogen through aromatase enzyme inhibition [30]. Moreover, high concentration of eurycomanone has inhibitory effect on phosphodiesterase [30].

It is worth mentioning that the IC_50_ of the MeCl and the diaion fractions (D1–D4) is less than that of the isolated pure compounds (Table 2). Hence, these fractions have better ROCK inhibitory potential than the isolated compounds (T1-8). Further studies are highly recommended to verify if this is due to the synergistic effects of the compounds in the mentioned fractions or there are much more potent compounds to be isolated from these fractions.

## 5. Conclusion

Our research revealed that the traditional use of* E. longifolia* as aphrodisiac and for male sexual disorders might be partially due to the ROCK-II inhibitory activity. To confirm our hypothesis, our future work is to study the in vivo aphrodisiac effect of the plant in animal model.

## Figures and Tables

**Table 1:** Effect of aqueous extract (AE), fractions and isolates on percentage inhibition of ROCK-II activity.

Treatment (s)	Concentrations (*µ*g/ml)	% of inhibition of ROCK-II ± SD
**Control**	*-*	*0*
**Vehicle control **	5%	2.06 ± 0.075
**AE**	10	81 ± 0.21*∗*
**AE**	1	62.8 ± 0.84*∗*
**AE**	0.1	16.3 ± 1.26*∗*
**AE**	0.01	3.18 ± 2.4
**MeCl**	10	*86.3*± 0.71*∗*
**MeCl**	1	76.8 ± 0.84*∗*
**MeCl**	0.1	46.9 ± 0.56*∗*
**MeCl**	0.01	17.2 ± 1.27*∗*
**D1**	10	*90.1*± 0.84*∗*
**D1**	1	76.3 ± 1.69*∗*
**D1**	0.1	55.8 ± 0.14*∗*
**D1**	0.01	21.1 ± 1.83*∗*
**D2**	10	*86.1*± 0.42*∗*
**D2**	1	74.04 ± 0.01*∗*
**D2**	0.1	49.65 ± 0.21*∗*
**D2**	0.01	18.9 ± 3.39*∗*
**D3**	10	*90.25*± 0.07*∗*
**D3**	1	80.15 ± 0.48*∗*
**D3**	0.1	61.8 ± 0.42*∗*
**D3**	0.01	17.9 ± 1.83*∗*
**D4**	10	*87.05*± 0.21*∗*
**D4**	1	74.05 ± 0.35*∗*
**D4**	0.1	46.1 ± 4.37*∗*
**D4**	0.01	14.5 ± 1.98*∗*
**T1**	10	60.3 ± 0.14*∗*
**T1**	1	27.35 ± 2.47*∗*
**T1**	0.1	13.3 ± 2.26*∗*
**T1**	0.01	2.78 ± 1.84
**T2**	10	82.1 ± 0.63*∗*
**T2**	1	68.5 ± 0.35*∗*
**T2**	0.1	33.2 ± 1.55*∗*
**T2**	0.01	10.62 ± 2.94*∗*
**T3**	10	77.1 ± 0.11*∗*
**T3**	1	66.15 ± 0.07*∗*
**T3**	0.1	25 ± 2.47*∗*
**T3**	0.01	10.1 ± 3.34*∗*
**T4**	10	76.2 ± 3.53*∗*
**T4**	1	54 ± 0.42*∗*
**T4**	0.1	25.7 ± 2.47*∗*
**T4**	0.01	6.13 ± 0.44*∗*
**T5**	10	74.5 ± 1.27*∗*
**T5**	1	55.3 ± 0.45*∗*
**T5**	0.1	23.6 ± 2.4*∗*
**T5**	0.01	8.1 ± 2.27*∗*
**T6**	10	78.3 ± 0.38*∗*
**T6**	1	57.1 ± 0.56*∗*
**T6**	0.1	26.4 ± 2.61*∗*
**T6**	0.01	5.4 ± 3.74
**T7**	10	74.1 ± 2.97*∗*
**T7**	1	49.6 ± 7.89*∗*
**T7**	0.1	19.4 ± 0.14*∗*
**T7**	0.01	5.85 ± 2.93
**T8**	10	71.4 ± 2.54*∗*
**T8**	1	46.5 ± 1.62*∗*
**T8**	0.1	17 ± 0.77*∗*
**T8**	0.01	4.06 ± 3.8
**Y-27632**	10	*89.9*± 0.42*∗*
**Y-27632**	1	68.15 ± 2.75*∗*
**Y-27632**	0.10	42.15 ± 2.19*∗*
**Y-27632**	0.01	22.5 ± 1.27*∗*

**AE: **aqueous extract;** D1**: water (100%); **D2:** methanol-water (50%); **D3**: methanol-water (75%); **D4**: methanol (100%) diaion fractions; **MeCl: **methylene chloride fraction; **T1: **stigmasterol; **T2: ***trans-*coniferyl aldehyde; **T3: **scopoletin; **T4: **eurycomalactone; **T6**: 6*α*- hydroxyeurycomalactone; **T6**: eurycomanone;** T7**: eurycomanol; **T8**: and eurycomanol-2-*O*-*β*-D-glucopyranoside.

*∗* Significant from Vehicle control at *P* < 0.0001

**Table 2:** IC_50_ of aqueous extract (AE) of *E. longifolia* root, its fractions, and its isolates expressed as mean ± SD. Assay was performed in triplicates

Sample	IC_50_ (ng/ml)
AE	651.1 ± 32.9*∗*
MeCl	162.8 ± 3.35*∗*
D1	105 ± 3.56*∗*
D2	153 ± 14.1#
D3	91.1 ± 6.63*∗*
D4	189.3 ± 21.9
T1	6141 ± 540*∗*
T2	358 ± 31.1*∗*
T3	525 ± 42.8*∗*
T4	794 ± 95.5*∗*
T5	829 ± 41*∗*
T6	677 ± 51.1*∗*
T7	1112 ± 73.7*∗*
T8	1441 ± 175*∗*
Y-27632	192 ± 8.37

IC50 values are mean ± SD. Statistical analysis was carried out by one-way ANOVA followed by Bonferroni post-hoc test. n=3

**∗**Significantly different from Y-27632 at *P* < 0.001

# Significant different from Y-27632 at *P* < 0.01

**AE: **aqueous extract;** D1**: water (100%); **D2:** methanol-water (50%); **D3**: methanol-water (75%); **D4**: methanol (100%) diaion fractions; **MeCl: **methylene chloride fraction; **T1: **stigmasterol; **T2: ***trans-*coniferyl aldehyde; **T3: **scopoletin; **T4: **eurycomalactone; **T6**: 6*α*- hydroxyeurycomalactone; **T6**: eurycomanone;** T7**: eurycomanol; **T8**: and eurycomanol-2-*O*-*β*-D-glucopyranoside.

## Data Availability

The data used to support the findings of this study are included within the article.

## Supplementary Materials

Supplementary Materials
^1^H NMR data of the isolated phytochemicals are presented in Tables S1. ^13^CNMR data of the isolated phytochemicals are presented in Tables S2.
