# Supplementary material for: Rho-Kinase II Inhibitory Potential of Eurycoma longifolia New Isolate for the Management of Erectile Dysfunction
Source: Evid Based Complement Alternat Med. 2019 May 15;2019:4341592. doi: 10.1155/2019/4341592 (PMC6541974; doi:10.1155/2019/4341592)
Supplement: Supplementary Materials — 1H NMR data of the isolated phytochemicals are presented in Tables S1. 13CNMR data of the isolated phytochemicals are presented in Tables S2. [file 4341592.f1.docx]

**Table S1. ^1^H NMR data of compounds T1- T8 (^1^H 400MHz; *δ* in ppm, *J* in Hz)**

| **Position** | **T1** | **T2** | **T 3** | **T 4** | **T 5** | **T 6** | **T 7** | **T 8** |
| --- | --- | --- | --- | --- | --- | --- | --- | --- |
| 1 | 2.23(*m*),2.97(*d,J*=12.8) |  |  | 4.04  (d, *J* = 1.3) | 3.98  (d, *J* = 1.2) | 4.34 (1H,s) | 3.52 (1H, s) | 4.21 (1H,d,*J*=8) |
| 2 | 1.77(*m*),2.13(*m*) | 6.99  (d, *J*=1.76) |  |  |  |  | 4.74 (1H, d) | 4.56(1H,m) |
| 3 | 3.51  (1H, m) | 3-OCH3  3.82 (s) | 6.11 (1H,d, *J*=9.4) | 6.04  (d, *J* = 2.5) | 6.14  (d, *J* = 2.5) | 5.99(1H, brs) | 5.21 (1H,s) | 5.79(1H,brs) |
| 4 |  |  | 7.62 (1H,d,*J*=9.4) |  |  |  |  |  |
| 5 |  | 6.88  (d, *J*=8.16) | 6.73 (1H,s) | 2.92  (dddq, *J* = 13,4.5,2,3,1) | 2.69  (dddq, *J* = 11, 2.3, 1.1) | 3.16 (1H,d) | 3.33 (1H,d) | 2.73  (1H,dd, *J*=13, 2.2) |
| 6a | 5.4  (1H, br.s) | 7.04 (dd, *J*=1.8,8.16) |  | 2.89 (dd, *J* = 14.1, 13.2) | 4.80  (dd, 11.3, 2.5) | 2.08 (1H,m) | 2.29 (1H,m) | 1.83 (1H,ddd, *J*=15,12.5,2) |
| 6b |  |  |  | 2.72 (s) |  | 2.50 (1H,m) | 2.50 (1H,m) | 2.08 (1H,ddd, *J*=15,12.5,2) |
| 7 |  | 7.31  (d, *J*=15.8) |  |  |  | 4.58 (1H,t) | 4.42(1H,d) | 5.12  (1H,dd,*J*=2.1,2.1) |
| 8 |  | 6.49 (dd, *J*=7.70,15.8) | 6.83 (1H,s) |  |  |  |  |  |
| 9 |  | 9.56  (d, *J*=7.70) |  | 1.81  (d, *J* = 3.3) | 1.79 (d, *J* = 3.5) | 3.34 (1H,s) | 2.54 (1H,s) | 3.45(1H,s) |
| 11 |  |  |  | 4.81  (ddd, *J* = 5.2,4.6,3.7) | 4.82  (ddd, *J* = 5.7, 4.6,3.8) |  |  |  |
| 12 |  |  |  | 4.32 (dd, *J* = 4.3,1.3) | 4.32 (dd, 4.6,1.2) | 4.57 (1H,s) | 4.54 (1H, brs) | 4.74(1H,s) |
| 13 |  |  |  | 2.91 (q, *J* = 7) | 2.91 (q, *J* = 7.2) |  |  |  |
| 14 |  |  |  | 2.96 (d, *J* = 1.3) | 2.94 (d, *J* = 1.1) |  |  |  |
| 15 |  |  |  |  |  | 3.72(1H,s) | 3.82(1H,s) | 3.98 (1H,s) |
| 17 |  |  |  |  |  | 1.93 (3H,s) | 1.61 (3H,s) | 1.61 (3H,s) |
| 18a | 1.27 (3H, s) |  |  |  |  | 4.58 (1H,d, , J=6. 28) | 4.46 (1H,d, J=6.24) | 5.07(1H,d, J=6.44) |
| 18b |  |  |  |  |  | 5.25 (1H, d, , J=6. 28) | 4.76 (1H,d, J=6.24) | 5.20(1H,d,J=6.44) |
| 19 | 1.66 (3H, s) |  |  |  |  | 1.11 (3H,s) | 1.07(3H,s) | 1.47 (3H, brs) |
| 21 | 0.71 (3H, d, *J*=5.4) |  |  |  |  |  |  |  |
| 22 | 5.15 (dd, 1H, *J*=8.3, 15.4) |  |  |  |  |  |  |  |
| 23 | 5.21 (1H, dd, *J*=8, 15.2) |  |  |  |  |  |  |  |
| 26 | 0.90 (3H, d, *J*=6.4) |  |  |  |  |  |  |  |
| 27 | 1.043 (3H, d, *J*=6.3) |  |  |  |  |  |  |  |
| 29 | 0.85 (3H, t, *J*=6.3) |  |  |  |  |  |  |  |
| 30a |  |  |  |  |  | 3.55(1H,d,*J*=8.6) | 3.54 (1H,brs) | 4.07(1H,d,*J*=8.1) |
| 30b |  |  |  |  |  | 3.67(1H,d,*J*=8.6) |  | 4.50(1H,d,*J*=8.1) |
| Me-4 |  |  |  | 1.94 (dd, *J* = 1.3,1) | 2.26 (dd, *J* = 1.3, 1.3) |  |  |  |
| Me-6 |  |  | 3.82(3H,s) |  |  |  |  |  |
| Me-8 |  |  |  | 1.56 (s) | 1.62 (s) |  |  |  |
| Me-10 |  |  |  | 1.26 (s) | 1.29 (s) |  |  |  |
| Me-13 |  |  |  | 1.12  (d, *J* = 7.2) | 1.13  (d, *J* = 7.4) |  |  |  |
| OH-1 |  |  |  | 4.62  (d, *J* = 1.5) | 4.65  (d, *J* = 1.1) |  |  |  |
| OH-6 |  |  |  |  | 4.04  (d, *J* = 2.8) |  |  |  |
| OH-11 |  |  |  | 3.00  (d, *J* = 5.4) | 3.03  (d, *J* = 5.7) |  |  |  |
| 1` |  |  |  |  |  |  |  | 5.20(1H,d,*J*=7.6) |
| 2` |  |  |  |  |  |  |  | 4.11(1H,dd,*J*=8.5,7.6) |
| 3` |  |  |  |  |  |  |  | 4.22 (1H,dd,*J*=8.5,8.7) |
| 4` |  |  |  |  |  |  |  | 4.39(1H,dd,*J*=8.7,8) |
| 5` |  |  |  |  |  |  |  | 3.98 (1H,dd, *J*=8, 5.2,2.3) |
| 6` |  |  |  |  |  |  |  | 4.39(1H,dd,*J*=11,5.2)  4.53(1H,dd,*J*=11,2.3) |

**Table S2. ^13^C-NMR data of the isolated compounds T1 - T8**

| **C** | **T1** | **T2** | **T3** | **T4** | **T5** | **T6** | **T7** | **T8** |
| --- | --- | --- | --- | --- | --- | --- | --- | --- |
| **1** | 37.26 | 126.6 |  | 81.53 | 80.9 | 82.84 | 82.64 | 83.77 |
| **2** | 31.91 | 109.4 | 162.93 | 198.29 | 198.08 | 197.62 | 71.63 | 82.35 |
| **3** | 71.82 | 146.9 | 108.3 | 125.66 | 125.77 | 125.28 | 125.94 | 124.45 |
| **4** | 44.22 | 148.9 | 144.62 | 162.54 | 165.34 | 163.12 | 134.43 | 134.9 |
| **5** | 140.76 | 114.9 | 111.7 | 49.78 | 56.72 | 41.19 | 40.4 | 40.88 |
| **6** | 121.72 | 124.0 | 145.62 | 35.98 | 69.78 | 25.09 | 24.92 | 25.17 |
| **7** | 31.88 | 153.1 | 151.27 | 206.65 | 207.78 | 70.92 | 70.74 | 71.29 |
| **8** | 31.68 | 126.4 | 103.12 | 51.2 | 49.22 | 51.62 | 51.7 | 52.43 |
| **9** | 50.7 | 193.6 | 149.99 | 48.43 | 48.14 | 46.55 | 46.85 | 47.38 |
| **10** | 36.52 |  | 111.16 | 47.08 | 47.95 | 45.25 | 41.64 | 42.11 |
| **11** | 21.09 |  |  | 62.29 | 69.14 | 108.2 | 108.31 | 109.36 |
| **12** | 39.69 |  |  | 83.7 | 83.55 | 79.6 | 79.68 | 80.77 |
| **13** | 44.32 |  |  | 32.27 | 31.89 | 146.56 | 146.25 | 147.9 |
| **14** | 56.87 |  |  | 52.96 | 53.46 | 78.45 | 78.25 | 79.13 |
| **15** | 24.37 |  |  | 177.35 | 176.81 | 75.14 | 75.68 | 76.04 |
| **16** | 28.9 |  |  | **CH3(13)**16.24 | 16.31 | 172.79 | 172.88 | 173.59 |
| **17** | 56.08 |  |  | **CH3(10)**11.41 | 12.95 | 22.82 | 21.44 | 20.91 |
| **18** | 40.49 |  |  | **CH3(4)**21.58 | 24.4 | 119.2 | 119.68 | 119.41 |
| **19** | 21.22 |  |  | **CH3(8)**23.17 | 22.73 | 10.01 | 10.21 | 10.43 |
| **20** | 138.32 |  |  |  |  |  |  | **C1'**106.07 |
| **21** | 129.28 |  |  |  |  |  |  | **C2'**75.89 |
| **22** | 45.84 |  |  |  |  |  |  | **C3'**78.37 |
| **23** | 25.41 |  |  |  |  |  |  | **C4'**71.66 |
| **24** | 12.05 |  |  |  |  |  |  | **C5'**78.25 |
| **25** | 29.7 |  |  |  |  |  |  | **C6'**62.41 |
| **26** | 19.82 |  |  |  |  |  |  |  |
| **27** | 19.4 |  |  |  |  |  |  |  |
| **28** | 18.89 |  |  |  |  |  |  |  |
| **29** | 12.25 |  |  |  |  |  |  |  |
| **30** |  |  |  |  |  | 66.57 | 66.69 | 67.6 |
| **3-OCH_3_** | 56.0 |  |  |  |  |  |  |  |
| **Molecular formula** | C_29_H_48_O |  | C_10_H_8_O_4_ | C_19_H_24_O_6_ | C_19_H_24_O_7_ | C_20_H_24_O_9_ | C_20_H_26_O_9_ | C_26_H_36_O_14_ |
